# Exploring the Relationship Between Trait Mindfulness and Interpersonal Sensitivity for Chinese College Students: The Mediating Role of Negative Emotions and Moderating Role of Effectiveness/Authenticity

**DOI:** 10.3389/fpsyg.2021.624340

**Published:** 2021-04-12

**Authors:** Xiaoqian Ding, Tian Zhao, Xiaoxi Li, Zirong Yang, Yi-Yuan Tang

**Affiliations:** ^1^College of Psychology, Liaoning Normal University, Dalian, China; ^2^Psychological Counseling Center, Dalian University of Foreign Languages, Dalian, China; ^3^Department of Gastroenterology, Affiliated Zhongshan Hospital of Dalian University, Dalian, China; ^4^Department of Psychological Sciences, Texas Tech University, Lubbock, TX, United States

**Keywords:** trait mindfulness, interpersonal sensitivity, negative emotions, emotional creativity, effectiveness, authenticity

## Abstract

**Background:** Interpersonal sensitivity is a prominent mental health problem facing college students today. Trait mindfulness is a potential positive factor that may influence interpersonal relationships. However, the precise relationship between trait mindfulness and interpersonal sensitivity remains elusive, which limits the optimization and further application of mindfulness-based intervention schemes targeting interpersonal sensitivity. This study aimed to explore (a) whether negative emotions mediate the relationship between trait mindfulness and interpersonal sensitivity and (b) whether the relationship among trait mindfulness, negative emotions, and interpersonal sensitivity is moderated by effectiveness/authenticity. We hypothesize that (a) negative emotions mediate the relationship between trait mindfulness and interpersonal sensitivity, and (b) effectiveness/authenticity moderates the indirect association between trait mindfulness and interpersonal sensitivity through negative emotions.

**Methods:** One thousand four hundred nineteen Chinese college students (1,023 females, 396 males), aged from 17 to 23 (SD = 0.86, mean = 18.38), participated in this study. Their trait mindfulness, negative emotions, the effectiveness/authenticity, and interpersonal sensitivity were measured using well-validated self-report questionnaires.

**Results:** Correlational analyses indicated that both trait mindfulness and effectiveness/authenticity were significantly and negatively associated with interpersonal sensitivity. Mediation analyses uncovered a partial mediating role of negative emotions in the relationship between trait mindfulness and interpersonal sensitivity. Moderated mediation analyses showed that in college students with high effectiveness/authenticity, the relationship between trait mindfulness and negative emotions was stronger, whereas the relationship between negative emotions and interpersonal sensitivity was weaker.

**Conclusion:** Negative emotion is a mediator of the relationship between trait mindfulness and interpersonal sensitivity, which in turn is moderated by effectiveness/authenticity. These findings suggest a potential mechanism through which trait mindfulness influences interpersonal sensitivity. Mindfulness-based interventions have the potential to decrease interpersonal sensitivity and offer a basis for predicting individual differences in response to mindfulness-based interventions among individuals.

## Background

Interpersonal sensitivity is a type of personality associated with low self-esteem and negative self-concept (Meisel et al., 2018). It is an undue and overawareness of the conduct and feelings of others and sensitivity to perceived criticism or rejection (Boyce et al., 1991). Thus, such sensitive individuals are highly alert to the expectations of others and afraid of being judged negatively and adjust their behaviors to minimize the risk of social exclusion. In other words, they often avoid behaviors that make them feel less confident (Boyce et al., 1991). Interpersonal sensitivity is a prominent mental health problem facing college students today (Zheng et al., 2017). Reportedly, the number of college students with a moderate or high intensity of interpersonal sensitivity is the second most prominent mental health symptoms after compulsion symptoms (Xu et al., 2011) and is significantly higher compared to other common psychological symptoms (Zheng et al., 2017). Additionally, interpersonal sensitivity is a psychological risk factor for infectious diseases and possibly cardiovascular diseases (Liu and Gu, 2015). It is also associated with or can be used as a predictor for many mental disorders and personality disorders, such as depression, paranoia, social phobia, and borderline personality disorder (Boyce et al., 1991; Zanarini and Frankenburg, 2001; Freeman et al., 2005; Kumari et al., 2012; Meisel et al., 2018). A 50-year community-based longitudinal observation in Sweden shows that the risk of developing psychosis is doubled among individuals initially assessed as sensitive or vulnerable to others (Bogren et al., 2010). Given the negative effects mentioned previously, exploring potential factors and mechanisms that contribute to interpersonal sensitivity is of theoretical and clinical importance.

Mindfulness refers to a non-reactive, non-judgmental, and present-centered awareness that acknowledges and accepts any feeling, thinking, or sensation as it is (Bishop et al., 2004). Theories of mindfulness suggest that it is like a character merit in constructive psychology that stands for a natural trait varying among people and is a state of consciousness that can be developed with mindfulness exercises (Davidson, 2010). Some studies show that mindfulness-based interventions can improve mindfulness, which contributes to reduced interpersonal distress and interpersonal sensitivity symptoms (Du et al., 2016; Qiu et al., 2017; Joss et al., 2020). Therefore, the correlation between trait mindfulness and interpersonal sensitivity is apparent.

However, the mediating mechanisms underlying this relationship (i.e., how does trait mindfulness relate to interpersonal sensitivity) and the moderating mechanisms (i.e., when is this relationship most effective) remain largely unknown. In this study, we aim to examine a conceptual model based on young adults, in which negative emotions mediate the relationship between trait mindfulness and interpersonal sensitivity; and effectiveness/authenticity (EA) moderates the indirect association between trait mindfulness and interpersonal sensitivity through negative emotions.

### Negative Emotions, Trait Mindfulness, and Interpersonal Sensitivity

The ability to regulate emotional experience is a basic human skill that contributes to interpersonal function, positive influence, and general well-being (Bullis et al., 2014). Negative emotions are closely related to maladaptive social interactions (Furr and Funder, 1998). Individuals with anxiety and depression often face more interpersonal difficulties (Epkins and Heckler, 2011; Hames et al., 2013). In a study of patients on methadone maintenance therapy, emotional factors such as depression and anxiety are risk factors for interpersonal sensitivity (Yang et al., 2015).

Mindfulness-based interventions directly affect the attention distribution of emotion regulation and improve negative emotions (Harvey et al., 2002; Tang et al., 2007; Ding et al., 2015; Du et al., 2016; Qiu et al., 2017). For instance, mindfulness effectively ameliorates persistent maladaptive cognitive content and affective symptoms associated with depression and anxiety (Ramel et al., 2004). As such, mindful individuals can better regulate their moods with emotional awareness and clarity, thereby suffering less from negative affect and related symptoms (Erismam and Roemer, 2010). In addition, trait mindfulness is negatively related to negative affectivity (Brown and Ryan, 2003) and contributes to the awareness and acceptance of negative emotions and thinking. Taken together, these studies indicate a relationship between higher trait mindfulness and fewer negative emotions.

Although it has not yet been tested, it is reasonable to speculate that negative emotions would play a mediating role in the relationship between trait mindfulness and interpersonal sensitivity. Therefore, one goal of the study is to test for the mediating role of negative emotions.

### EA, Trait Mindfulness, and Interpersonal Sensitivity

Although trait mindfulness may influence interpersonal sensitivity *via* the mediating effect of negative emotions, individuals with the same level of mindfulness do not necessarily have the same level of negative emotions and interpersonal sensitivity. The heterogeneity of outcomes may be due to other individual characteristics.

Emotional creativity refers to the ability to honestly perceive and convey fresh and active mixed emotions (Averill and Thomas-Knowles, 1991) and to express emotions to meet the needs of individuals or interpersonal situations (Gutbezahl and Averill, 1996), which focuses on the internal emotions generated during interpersonal interaction. EA, one indicator of emotional creativity, refers to the skill to express emotions freely and honestly (Averill, 1999; Ivcevic et al., 2007). When faced with stressful situations, individuals with high emotional creativity prefer strategies of self-control, positive reappraisal, planned problem solving, and seeking social support (Averill, 1999), which enable better adaptation to the environment. More importantly, EA is inversely correlated with escape-avoidance coping styles (Averill, 1999), which is one of the behavioral characteristics of interpersonal sensitivity. As an indicator of emotional creativity, the relationship between EA and emotion is self-evident.

Therefore, it is possible that EA may moderate the indirect relationship between trait mindfulness and interpersonal sensitivity.

### The Present Study

**Figure 1:**
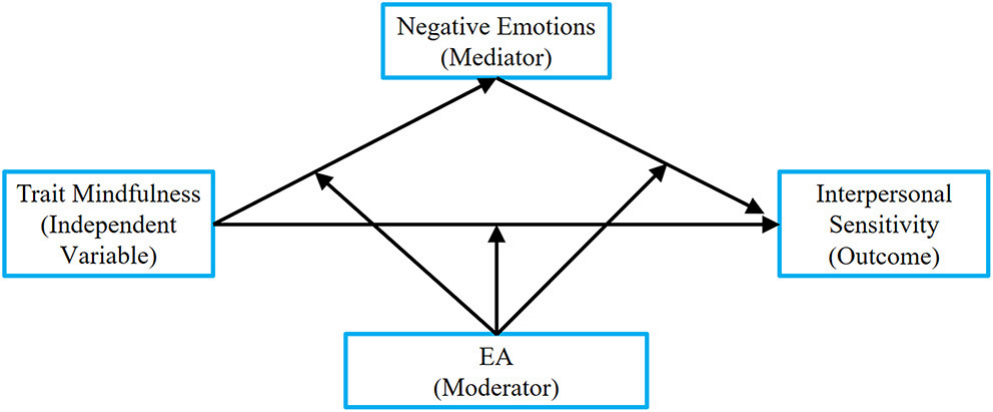
The conditional process model.

A conceptual model underlying the protective role of trait mindfulness in interpersonal sensitivity was tested. Specifically, this study aimed to examine (a) whether negative emotions mediate the relationship between trait mindfulness and interpersonal sensitivity and (b) whether EA moderates the indirect connection between trait mindfulness and interpersonal sensitivity through negative emotions. Taken together, these two questions constitute a moderated mediation model. This integrated model can simultaneously address questions regarding mediation (i.e., how trait mindfulness relates to interpersonal sensitivity) and moderation (i.e., when and for whom is the connection weakest or strongest), see Figure 1. Based on previous studies, we propose two hypotheses:

**Hypothesis 1**. There would be a negative correlation between trait mindfulness and negative emotions, and a positive correlation between negative emotions and interpersonal sensitivity, and that negative emotions would mediate the relationship between trait mindfulness and interpersonal sensitivity.
**Hypothesis 2**. EA would negatively predict interpersonal sensitivity. Furthermore, the indirect association between trait mindfulness and interpersonal sensitivity through negative emotions would vary as a function of EA.

## Methods

### Participants and Procedures

All materials and procedures were reviewed and approved by the Institutional Review Board of Liaoning Normal University. This study was conducted following the regulations that have been established for human subject protection. A total of 1,528 college students were randomly selected to complete online questionnaires through the psychological test system for college students in 2019. One thousand four hundred nineteen college students with ages ranging from 17 to 23 years (SD = 0.86 years, mean = 18.38 years) and without prior mindfulness practice experience (1,023 females, 396 males) returned valid data. The participation was voluntary, and participants were free to discontinue their participation at any time during the study. All participants were informed about the effectiveness, independence, and integral nature of all the answers, and all participants received a gift as a reward for their participation.

### Measures

#### Mindful Attention Awareness Scale

To evaluate individual differences in trait mindfulness, we used the Mindful Attention Awareness Scale (MAAS) (Brown and Ryan, 2003), which was translated and adapted into Chinese by Deng et al. (2012). MAAS includes 15 items (e.g., I find it hard to keep concentrated on what is happening). Participants rated each item on a 6-point scale from 1 = almost always to 6 = almost never, with a larger score indicating more mindfulness. MAAS showed high reliability (Cronbach α = 0.91) in this study.

#### Profile of Mood States

To examine transient and definite mood states, we used the Profile of Mood States (POMS) (Mcnair et al., 1971), which was translated into Chinese and has demonstrated high reliability (Cronbach α = 0.95) (Wang et al., 2000). This 65-item scale focuses on six mood factors: tension–nervousness, depression–dispiritedness, anger–unfriendliness, vigor–energy, tiredness–inertia, and confusion–bewilderment. Participants rated each item from 0 = not at all to 4 = extremely. The total mood disturbance (TMD) is scored as the sum of scores of five of the six mood factors (no vigor–energy) and added by 100. The TMD score is an overall indicator of negative emotion, and a higher score implies worse mood state.

#### Symptom Checklist 90

Symptom Checklist 90 (SCL-90) (Derogatis et al., 1973) assesses mental health symptoms and consists of 90 items and 9 subscales. One of them, a nine-item (e.g., “feeling un-understood or unsympathetic”) interpersonal sensitivity factor, was used to assess an individual's discomfort and inferiority in interpersonal communication, especially when compared with others. Participants rated each item from 1 = “perceived absence of the problem” to 5 = “perceived severity and frequency of the symptoms.” The edition used here was revised by Wang (1984). SCL-90 showed high reliability (Cronbach α = 0.85) in this study.

#### Emotional Creativity Inventory

This self-report scale consists of 30 questions and is divided into three subscales (Averill and Thomas-Knowles, 1991). The EA subscale (e.g., “sometimes my emotional experience and emotional expression can help me improve my interpersonal relationship”) was used to assess the self-perception of EA. Participants rated each item from 1 = total nonconformity to 5 = total conformity. In this study, we used the edition revised by Wang and Yan (2017). A high reliability was detected for each subscale (EA: Cronbach α = 0.81; preparedness: Cronbach α = 0.76; novelty: Cronbach α = 0.89).

### Statistical Analyses

Descriptive data and correlation matrix were computed first, followed by a mediation analysis, which was computed using a four-step method (MacKinnon, 2008). Next, we explored if EA moderated the mediation. Moderated mediation is commonly adopted to test if the magnitude of a mediation impact relies on the value of a moderator (Muller et al., 2005). The moderated mediation was analyzed using Hayes's (2013) PROCESS macro (model 59). Continuous data were normalized and used to compute the interaction terms.

## Results

We took three steps to answer the two study questions: whether negative emotions mediate the relationship between trait mindfulness and interpersonal sensitivity and whether the indirect path between trait mindfulness and interpersonal sensitivity is moderated by EA.

### Preliminary Analysis

Correlations among trait mindfulness, negative emotions, interpersonal sensitivity, EA, preparedness, and novelty were analyzed. Means, standard deviations (SDs), and zero-order correlations of all parameters are listed in Table 1. As expected, college students with more trait mindfulness or less negative emotions were less prone to interpersonal sensitivity. Additionally, students with higher trait mindfulness had fewer negative emotions. Likewise, those with more EA had less interpersonal sensitivity, as well as fewer negative emotions and higher trait mindfulness. In addition, preparedness was not associated with interpersonal sensitivity (*p* = 0.11), and novelty was not associated with trait mindfulness (*p* = 0.06).

**Table 1:** Descriptive statistics and related analysis results of variables.

**Variables**	**M**	**SD**	**1**	**2**	**3**	**4**	**5**	**6**
1. Trait mindfulness (MAAS)	62.38	13.533	1					
2. Negative emotions (POMS)	140.43	33.615	−0.426^**^	1				
3. Interpersonal sensitivity (SCL-90)	13.90	5.339	−0.233^**^	0.314^**^	1			
4. EA (ECI)	26.78	4.972	0.289^**^	−0.206^**^	−0.151^**^	1		
5. Preparedness (ECI)	18.41	3.537	0.279^**^	−0.279^**^	−0.042	0.403^**^	1	
6. Novelty (ECI)	37.85	9.294	−0.050	0.252^**^	0.137^**^	0.380^**^	0.285^**^	1

*N = 1,419. ECI, Emotional Creativity Inventory; M, mean*.

* *p < 0.05*;

** *p < 0.01*.

### Testing for Mediation Effect

To test hypothesis 1, we adopted MacKinnon's (2008) four-step method to examine the mediation effect, which included testing for significant correlations (a) between trait mindfulness and interpersonal sensitivity, (b) between trait mindfulness and negative emotions, (c) between negative emotions and interpersonal sensitivity after controlling for trait mindfulness, and (d) a significant coefficient for the indirect path between trait mindfulness and interpersonal sensitivity through negative emotions. The bias-corrected percentile bootstrapping was applied to determine if the last requirement was met.

Multiple regression analysis demonstrated that mindfulness was significantly associated with interpersonal sensitivity (*b* = −0.23, *p* < 0.001, model 1 of Table 2) and negative emotions (*b* = −0.43, *p* < 0.001, model 2). Additionally, after controlling for mindfulness, negative emotions were highly associated with interpersonal sensitivity (*b* = 0.26, *p* < 0.001, model 3). Finally, the bias-corrected percentile bootstrapping revealed a significant indirect effect (i.e., mediation effect) of mindfulness on interpersonal sensitivity through negative emotions [*ab* = −0.11, SE = 0.02, 95% confidence interval = (−0.15, −0.08)], which accounted for 48% of the total effect. Overall, the four conditions of establishing a mediation effect were all met, supporting hypothesis 1.

**Table 2:** Testing the mediation effect of mindfulness on interpersonal sensitivity.

	**Model 1 (interpersonal sensitivity)**		**Model 2 (negative emotions)**		**Model 3 (interpersonal sensitivity)**	
**Predictors**	***b***	***t***	***b***	***t***	***b***	***t***
Trait mindfulness	−0.23	−9.04^***^	−0.43	−17.73^***^	−0.12	−4.39^***^
Negative emotions					0.26	9.48^***^
*R*^2^	0.05		0.18		0.11	
*F*	81.64^***^		314.37^***^		88.28^***^	

*N = 1,419. Each column is a regression model that forecasts the condition at the top of the column (the same below)*.

*** *p < 0.001*.

### Testing for Moderated Mediation

To examine hypothesis 2 (Figure 1), we used the PROCESS macro (model 59) (Hayes, 2013) to examine the moderated mediation. Specially, parameters of three regression models were evaluated. Model 1 focused on the moderating effect of EA on the relationship between trait mindfulness and interpersonal sensitivity. Model 2 focused the moderating impact of EA on the relationship between trait mindfulness and interpersonal sensitivity. Model 3 focused on the moderating effect of EA on the relationship between trait mindfulness and negative emotions, as well as on the relationship between negative emotions and interpersonal sensitivity. All three models are specified in Table 3.

**Table 3:** Testing the moderated mediation effect of trait mindfulness on interpersonal sensitivity.

	**Model 1 (interpersonal sensitivity)**		**Model 2 (negative emotions)**		**Model 3 (interpersonal sensitivity)**	
**Predictors**	***b***	***t***	***b***	***t***	***b***	***t***
Trait mindfulness	−0.21	−7.74^***^	−0.41	−16.60^***^	−0.11	−3.88^***^
EA	−0.09	−3.44^***^	−0.11	−4.39^***^	−0.07	−2.50^*^
Trait mindfulness × EA	−0.01	−0.74	−0.10	−5.89^***^	0.00	0.02
Negative emotions					0.26	9.12^***^
Negative emotions × EA					−0.06	−2.68^**^
*R*^2^	0.06		0.21		0.12	
*F*	31.38^***^		124.21^***^		38.43^***^	

*N = 1,419*.

* *p < 0.05*.

** *p < 0.01*.

*** *p < 0.001*.

Moderated mediation was constructed when both or either of the following conditions was met (Muller et al., 2005; Hayes, 2013): EA moderated (a) the route between trait mindfulness and negative emotions (first stage) and/or (b) the route between negative emotions and interpersonal sensitivity (second stage).

Model 1 revealed a major impact of trait mindfulness on interpersonal sensitivity (*b* = −0.21, *p* < 0.001), which was not moderated by EA (*b* = 0.01, *p* > 0.05, Table 3). Model 2 uncovered a main effect of trait mindfulness on negative emotions (*b* = −0.41, *p* < 0.001), which was moderated by EA (*b* = −0.10, *p* < 0.001). Model 3 indicated a significant effect of negative emotions on interpersonal sensitivity (*b* = 0.26, *p* < 0.001), which was moderated by EA (*b* = −0.06, *p* < 0.01).

**Figure 2:**
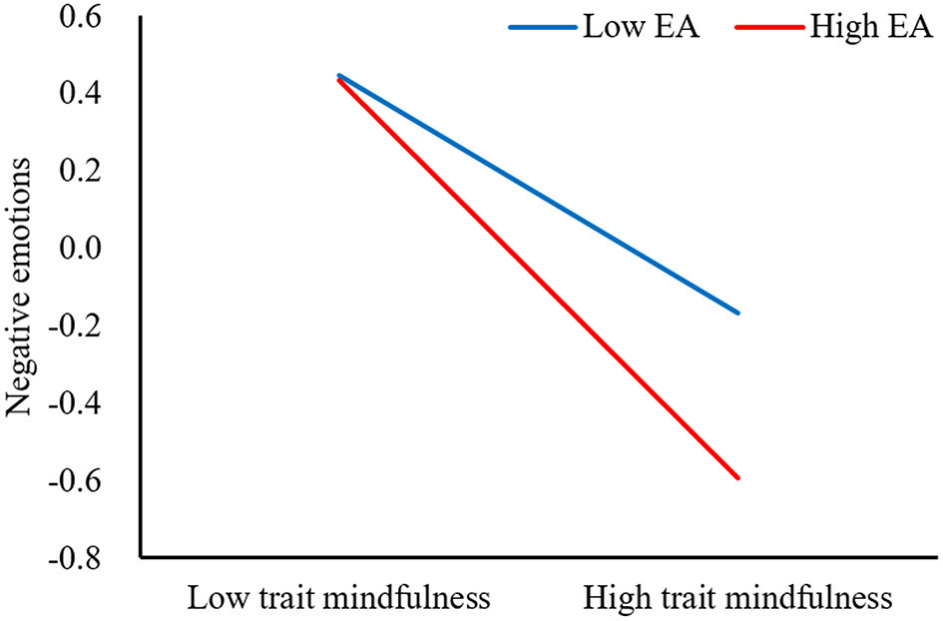
Effects of trait mindfulness and EA on negative emotions. Two levels of EA graphed include one standard deviation above and below the mean, respectively. The graph is for description only. All inferential analyses keep continuous data of trait mindfulness and EA (the same below).

**Figure 3:**
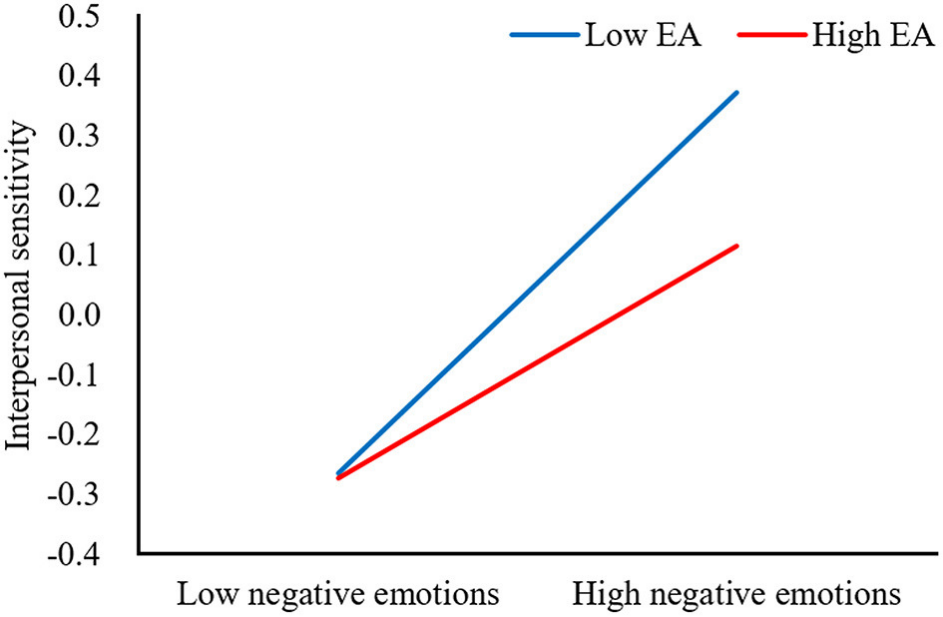
Effects of negative emotions and EA on interpersonal sensitivity.

We visualized the relationships between negative emotions and trait mindfulness in Figure 2 and between interpersonal and negative emotions in Figure 3 both at high and low (1 SD above and below the mean, respectively) levels of EA. First, simple slope tests indicated that compared to college students with low levels of EA, for college students with high levels of EA, higher levels of trait mindfulness were associated with lower levels of negative emotions (high EA: *b*_simple_ = −0.52, *p* < 0.001; low EA: *b*_simple_ = −0.31, *p* < 0.001; Figure 2). In other words, compared to the low-EA group, the effect of trait mindfulness on the negative emotion was stronger in the high-EA group. Second, simple slope tests revealed that negative emotions were significantly associated with interpersonal sensitivity for college students with both high and low levels of EA (high EA: *b*_simple_ = 0.24, *p* < 0.001; low EA: *b*_simple_ = 0.35, *p* < 0.001). But compared to the low-EA group, negative emotions had less effect on the interpersonal sensitivity in the high-EA group.

After incorporating gender into the model to test whether gender differences in EA affected its moderating effect, we found that the path “trait mindfulness × EA × gender” from trait mindfulness to negative emotions was not significant (*p* = 0.51), and the path “negative emotions × EA × gender” from negative emotions to interpersonal sensitivity was not significant (*p* = 0.26), indicating that there was no significant difference in EA between participants with different gender. Future research can expand the scope of sample selection to further assess the role of gender in these relationships.

## Discussion

Researchers of this study constructed a moderated mediation model to investigate if trait mindfulness was indirectly associated with interpersonal sensitivity *via* negative emotions and if this indirect association was moderated by EA. Results suggested that the effect of trait mindfulness on interpersonal sensitivity can be partially explained by negative emotions. In other words, trait mindfulness negatively predicted negative emotions, which in turn positively predicted interpersonal sensitivity. Furthermore, this indirect relationship was moderated by EA in the two-stage mediation, such that the path from trait mindfulness to negative emotions was stronger in the case of higher EA, and the path from negative emotions to interpersonal sensitivity was weaker in the case of higher EA. These results suggested that prevention and intervention strategies that seek to reduce interpersonal sensitivity through reducing negative emotion may be more effective among college students with stronger EA, whereas such strategies may not be as strong in college students with low EA. Each of the hypotheses is discussed below following the moderated mediation model of trait mindfulness and interpersonal sensitivity.

### Mediating Role of Negative Emotions

We considered the potential link between trait mindfulness and interpersonal sensitivity and tested the mediating role of negative emotions in this relationship. The results showed that trait mindfulness is negatively related to negative emotions, whereas negative emotions in turn are positively related to interpersonal sensitivity. Based on these findings, it is highly possible that low-level negative emotions can be one explanation for why college students with high trait mindfulness are less hypersensitive to interpersonal relationships. Relatedly, these findings may also suggest a putative mechanism by which mindfulness training alleviates interpersonal sensitivity in college students and provides theoretical support for the effect of mindfulness training in reducing interpersonal sensitivity (Du et al., 2016; Qiu et al., 2017).

In addition to the general mediation finding, the individual associations in the mediation models are also worth highlighting. At the first stage of mediation (i.e., trait mindfulness → negative emotions), our findings indicate that higher trait mindfulness is related to less negative emotions, which is consistent with prior literature showing the critical role of trait mindfulness in emotions (Harvey et al., 2002; Brown and Ryan, 2003; Ramel et al., 2004). College students with higher trait mindfulness are less likely to experience negative emotions and thereby would be unlikely to become hypersensitive to interpersonal relationships. At the second stage of mediation (i.e., negative emotions → interpersonal sensitivity), the result reveals that negative emotions are positively associated with interpersonal sensitivity, which is consistent with prior literature showing that those with higher degree of negative emotions reportedly have higher interpersonal sensitivity (Yang et al., 2015).

### Moderating Role of EA

Our second objective was to clarify if EA predicts interpersonal sensitivity and, more importantly, if EA moderates the indirect connection between trait mindfulness and interpersonal sensitivity. Results demonstrated that EA indeed predicted interpersonal sensitivity, such that individuals with lower EA are more likely to be more sensitive to interpersonal relationships.

Moreover, EA moderates both the paths between trait mindfulness and negative emotions (first stage) and between negative emotions and interpersonal sensitivity (second stage). Trait mindfulness is related more significantly with negative emotions among college students with high EA. Additionally, the relationship between negative emotions and interpersonal sensitivity is less significant among college students with high EA.

Altogether, by integrating the EA as a moderator, this study reveals the previously overlooked effects when moderation analysis was not used. Moreover, the moderated mediation model is theoretically more mature and more powerful in forecasting than the mediation model.

### Practical Implications

First, this study showed that people with higher levels of mindfulness have fewer negative emotions and lower interpersonal sensitivity, whereas negative emotions are positively correlated with interpersonal sensitivity. This is of great clinical significance to the development and implementation of prevention and intervention programs to reduce interpersonal sensitivity, as considering these individual difference factors is critical for accurately evaluating and enhancing the efficacy of prevention and intervention programs (Tang and Braver, 2020). For instance, trait mindfulness and negative emotions can be used as potential targets for intervention in young people with high interpersonal sensitivity, potentially through mindfulness training that enhances emotional regulation to reduce negative emotions, thereby leading to reduction in interpersonal sensitivity. On the other hand, emotional regulation training alone can also be helpful if it is effective at reducing negative emotions.

Second, the moderating role of EA should be emphasized. In particular, higher EA means that negative emotions would have a weaker impact on exacerbating interpersonal sensitivity, but trait mindfulness would have a stronger impact on modulating negative emotions. Thus, a mindfulness-based intervention targeting interpersonal sensitivity may be more effective for people with high EA, as both trait mindfulness and negative emotions would improve with the training.

Third, perfectionism has been divided into six dimensions by some researchers. Among them, personal standards and organization were regarded as positive (adaptive) perfectionism, whereas concern over mistakes, parental expectations, parental criticism, and doubts about actions were regarded as negative (maladaptive) perfectionism (Frost et al., 1990; Rice et al., 1998; Dunkley et al., 2006). Negative perfectionism is common among college students all over the world (Grzegorek et al., 2004; Rice and Ashby, 2007), and it is particularly common in or closely related to interpersonal sensitivity symptoms (Kang and Chen, 2012; Kumari et al., 2012). Therefore, a more detailed understanding of the factors that reduce interpersonal stress and alleviate interpersonal sensitivity symptoms may have important theoretical and clinical implications for reducing negative perfectionism tendency in college students, helping them to become more flexible in the face of problems.

Fourth, although studies have found the negative effects of interpersonal sensitivity on interpersonal relationships in different cultural environments, as well as its correlation with psychological or emotional problems such as depression and anxiety (Liu and Gu, 2015), these negative effects may be particularly important in collectivist cultures. In the collectivist culture, people are more closely connected with each other, attach more importance to their relationship with other people, and pay attention to the harmony among group members (Hofstede and Bond, 1984). Tong (2010) points out that an important Chinese cultural significance of psychological disorder lies in the appearance of interpersonal problems and the destruction of interpersonal relations. Therefore, under collectivist culture, alleviating the negative impact of interpersonal sensitivity may be particularly useful for preventing and improving psychological problems.

### Limitations and Future Directions

First, this study employed a cross-sectional design, which is not optimal for ascertaining causal inference. Future longitudinal studies should be designed to further explore the causal relationship between trait mindfulness and interpersonal sensitivity. Second, the data were collected only through self-reported questionnaires. Among the different mindfulness scales, there are differences in definition and context of the construct of mindfulness (Grossman, 2008, 2011; Grossman and Van Dam, 2011). The MAAS used in this study has many advantages, such as its validity, reliability, and stability for assessing levels of mindfulness (Phang et al., 2016) in a Chinese college population (Deng et al., 2012) and that it is simple and convenient for collecting a large sample data (Soler et al., 2012). However, future investigators need to further explore the mechanism underlying the effects of mindfulness on interpersonal sensitivity by comparing populations with and without mindfulness experience. Third, this study was based on a community of college students, and thus, the results should not be generalized to other samples. Future research could benefit from expanding the range of sample selection to community populations. In addition, the relationship in this study may be bidirectional. In the future, cross-lagged causality analysis can be carried out by means of repeated measurements to draw a more accurate conclusion.

## Conclusions

Trait mindfulness can exert a positive impact on interpersonal sensitivity through interacting with negative emotions. Moreover, EA moderates the relationship between trait mindfulness and negative emotions, as well as the relationship between negative emotions and interpersonal sensitivity. Importantly, this relationship between negative emotions and interpersonal sensitivity is less pronounced in individuals with high EA. That is, when college students have stronger EA, trait mindfulness has a stronger protective effect against negative emotions, while the deleterious impact of negative emotions on interpersonal sensitivity is attenuated.

## Data Availability Statement

The raw data supporting the conclusions of this article will be made available by the authors, without undue reservation.

## Ethics Statement

The studies involving human participants were reviewed and approved by Human Research Committee of Liaoning Normal University (LL2020003). Written informed consent to participate in this study was provided by the participants' legal guardian/next of kin.

## Author Contributions

XD designed the study, collected the data, analyzed and interpreted data, and participated in writing up the manuscript. TZ analyzed data and drafted the manuscript. XL and ZY collected the data and assisted paper writing. Y-YT conceived the idea, and participated in writing up and revising the manuscript. All authors read and approved the final manuscript.

## Conflict of Interest

The authors declare that the research was conducted in the absence of any commercial or financial relationships that could be construed as a potential conflict of interest.
